# A Nomogram Based on Hematological Parameters and Clinicopathological Characteristics for Predicting Local–Regional Recurrence After Breast-Conserving Therapy

**DOI:** 10.3389/fonc.2022.861210

**Published:** 2022-07-19

**Authors:** Luhao Sun, Wei Zhao, Fukai Wang, Xiang Song, Xinzhao Wang, Chao Li, Zhiyong Yu

**Affiliations:** Breast Cancer Center, Shandong Cancer Hospital and Institute, Shandong First Medical University and Shandong Academy of Medical Sciences, Jinan, China

**Keywords:** breast-conserving therapy, local–regional recurrence, hematological parameters, clinicopathological characteristics, nomogram, predicting model

## Abstract

**Objectives:**

The aim of this study was to identify the factors for local–regional recurrence (LRR) after breast-conserving therapy (BCT). We established a practical nomogram to predict the likelihood of LRR after BCT based on hematological parameters and clinicopathological features.

**Methods:**

A retrospective analysis was performed on 2,085 consecutive breast cancer patients who received BCT in Shandong Cancer Hospital from 2006 to 2016, including 1,460 patients in the training cohort and 625 patients in the validation cohort. Univariate and multivariate analyses were performed based on hematological parameters (fibrinogen, platelets, mean platelet volume, neutrophils, monocytes, and lymphocytes) and clinicopathological characteristics to identify the independent factors for LRR. Subsequently, a nomogram for predicting LRR was established by logistic regression analysis. The nomogram was validated in 625 patients in the validation cohort.

**Results:**

During the median follow-up period of 66 months, 44 (3.01%) patients in the training cohort and 19 (3.04%) patients in the validation cohort suffered from LRR. Multivariate analysis showed six independent factors related to LRR, including molecular subtype, pathological N stage, re-resection, radiotherapy or not, platelet count*MPV*fibrinogen (PMF), and neutrophil count/lymphocyte count ratio (NLR). Six variables were entered into logistic regression to establish the nomogram for predicting LRR. The nomogram of LRR showed excellent discrimination and prediction accuracy. The area under the receiver operating characteristic curve (AUC) was 0.89 (*p* < 0.001, 95% CI = 0.83, 0.95) in the training cohort and 0.88 (*p* < 0.001, 95% CI = 0.8, 0.96) in the validation cohort. Calibration curves for the prediction model in the training and validation cohorts both demonstrated satisfactory consistency between the nomogram-predicted and actual LRR.

**Conclusion:**

The combination of hematological parameters and clinicopathological characteristics can predict LRR after BCT. The predictive nomogram based on preoperative and postoperative indicators of BCT might serve as a practical tool for individualized prognostication. More prospective studies should be performed to verify the model.

## Introduction

Breast cancer is the most common malignancy in women and is also the main cause of female death (1). Multiple prospective randomized clinical trials have confirmed that breast-conserving therapy (BCT) plus radiotherapy is similar to mastectomy in terms of disease control and long-term overall survival (2–4). The BCT ratio in European and American countries has exceeded 60%. Although the BCT ratio in China is increasing, it is still at a low level, at only 20%–30%. The main reason is that a number of Chinese patients believe that BCT carries a risk of LRR compared to mastectomy. Therefore, it is necessary to study the LRR of the breast-conserving population in China. In this study, hematological parameters were innovatively added to predict LRR after BCT. Some previous studies have pointed out that hematological parameters (such as neutrophil count/lymphocyte count, lymphocyte count/monocyte count, and platelet count/lymphocyte count) have a satisfactory predictive effect on the recurrence of a few cancers, such as gastric cancer and bladder cancer (5–7). Therefore, we combined hematological parameters and clinicopathological features to predict the recurrence of breast cancer after BCT, and established a prediction model. Balancing survival and breast aesthetics, BCT has become the preferred local treatment for early invasive breast cancer (8). However, patients who received BCT and postoperative radiotherapy still suffered from LRR (3%–5%) in 10 years. Previous studies have found that clinicopathological characteristics (such as young age of onset, no radiotherapy, high nuclear grade, tumor stage, and molecular subtype) are factors for LRR after BCT (9, 10). For molecular subtype, according to the CSCO guidelines: Luminal A: HER-2 (−), ER (+), PR (+) and high expression, Ki67 low expression. Luminal B: HER-2 (−), ER (+), PR (−) or low expression, Ki67 high expression.

Hematological parameters (such as fibrinogen and platelets) have potential effects on the occurrence and development of tumors. Previous studies have reported that fibrinogen and platelets have synergistic effects in protecting tumor cells from NK cells (11, 12). Satoshi Takagi reported that platelets could promote the interaction between aggrus/podoplanin and CLEC-2 to promote tumor growth and metastasis (13). It also shown that platelets could promote immune escape adaptive immune responses by increasing the expression of PD-L1 in cancer cells (14). The mean platelet volume (MPV) level reflects the activity of platelets, which are elevated in patients with myocardial infarction and cancer (15). The tumor-induced systemic inflammatory response (SIR) can inhibit the function of T-cell immune monitoring and the immune response, causing tumor development and metastasis (16, 17). Inflammatory factors (neutrophils, monocytes, and lymphocytes) and platelets can be used to evaluate the host’s antitumor immune response and effectively predict the prognosis of cancer (18). Since tumor-associated inflammation is a basic component of tumor microenvironment, it may affect the prognosis of tumor. In a clinical setting, the detection of elevated inflammatory factors in the systemic circulation is widely considered to be a prognostic factor for many malignancies (19).

Therefore, this retrospective study was performed for two purposes: the first was to identify the factors related to the LRR of breast cancer treated by BCT, and the second was to establish a nomogram for predicting LRR after BCT by clinicopathological characteristics and hematological parameters.

## Materials and Methods

### Patient Population

The study retrospectively investigated the relationship between hematological parameters, clinicopathological features, and LRR at Shandong Cancer Hospital from 2006 to 2016. The eligibility criteria were as follows: (1) female patients with invasive carcinoma or ductal carcinoma *in situ* by pathology; (2) all patients were treated with BCT; (3) chemotherapy and radiotherapy were not received before the operation; (4) patients did not receive other anticancer treatment or blood transfusion before blood examination; and (5) all patients completed the analysis of hematological parameters after entering the hospital to the day before the operation.

**Figure 1:**
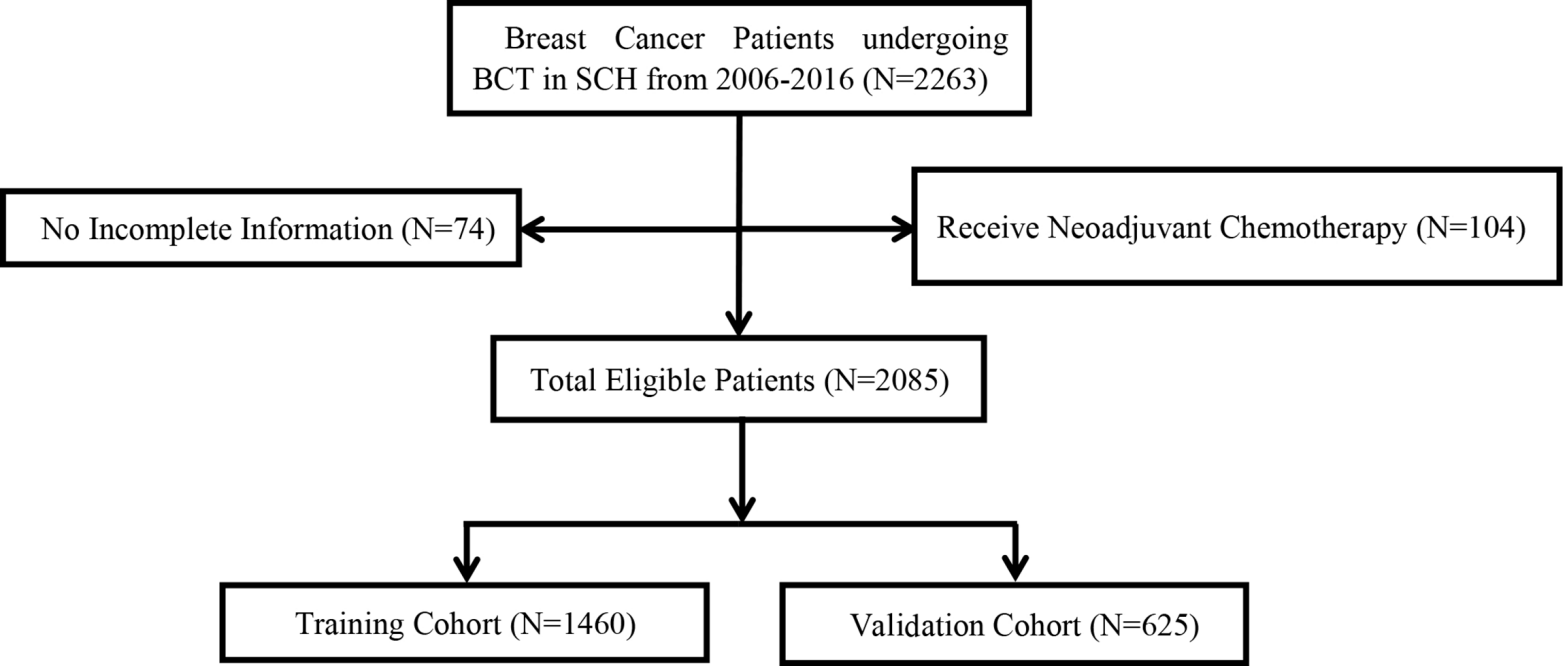
Consort diagram for the study cohort. BCT, breast cancer therapy; SCH, Shandong cancer hospital; LRR, local–regional recurrence.

Among all patients who received BCT from 2006 to 2016, 74 patients who had data loss and 104 patients who received neoadjuvant chemotherapy were excluded. Finally, 2,085 patients selected for the study were randomly divided into a training cohort (1,460) and a validation cohort (625) according to a 7:3 ratio (**Figure 1**).

The selected clinicopathological characteristics included the following: age of onset, menstrual status, pathological stage, presence of carcinoma *in situ*, molecular subtype, nuclear grade, re-resection, pathological T stage, pathological N stage, axillary surgery type, hormone receptor status, HER-2 status, chemotherapy, radiotherapy, and endocrine treatment, which are summarized in **Table 1**. The pathological stage was in accordance with the American Joint Commission on Cancer (AJCC) 7th edition staging standard. Histological grade was determined according to the World Health Organization (WHO) classification system.

**Table 1:** The basic information, tumor characteristics, and treatment methods of breast cancer patients receiving BCT.

Characteristic	Training cohort		Validation cohort	
	Total	LRR (%)	Total	LRR (%)
Age				
≤45	708	28 (3.9)	295	11 (3.7)
>45	752	16 (2.1)	330	8 (2.4)
Menopausal status				
Premenopausal	955	34 (3.6)	372	15 (4.0)
Postmenopausal	505	10 (2.0)	253 (33.4)	4 (1.6)
Nuclear grade				
I	220	2 (0.9)	112	3 (2.6)
II	731	17 (2.3)	292	10 (3.4)
III	443	23 (5.2)	177	6 (3.4)
Unknown	66	2 (3.0)	4	0 (0.0)
Pathologic T stage				
T1	1,041	34 (3.3)	388	11 (2.8)
T2	333	8 (2.4)	170	6 (3.5)
T3	10	0 (0.0)	3	0 (0.0)
Carcinoma *in situ*	76	2 (2.6)	64	2 (3.1)
Pathologic N stage				
N0	1,136	16 (1.4)	492	6 (1.2)
N1	276	16 (5.8)	117	8 (6.8)
N2	40	8 (20.0)	11	2 (18.2)
N3	8	4 (50.0)	5	3 (60.0)
Pathologic stage				
0	67	2 (3.0)	29	3 (10.3)
I	797	9 (1.1)	382	10 (2.6)
II	534	21 (3.9)	203	5 (2.5)
III	62	12 (19.4)	11	1 (9.1)
With carcinoma *in situ*				
Yes	318	21 (6.6)	189	10 (5.3)
No	1,142	23 (2.0)	436	9 (2.1)
Re-resection				
Yes	427	18 (4.2)	226	8 (3.5)
No	1,033	26 (2.5)	399	11 (2.8)
Molecular subtype				
Luminal A	707	7 (1.0)	298	4 (1.3)
Luminal B	114	3 (2.6)	48	1 (2.1)
HER-2 positive	504	20 (4.0)	226	7 (3.1)
TNBC	135	14 (10.4)	53	7 (13.2)
ER status				
Positive	1,140	19 (1.7)	403	12 (3.0)
Negative	320	25 (7.8)	222	7 (3.2)
PR status				
Positive	1,065	17 (1.6)	426	8 (1.9)
Negative	395	27 (6.8)	199	11 (5.5)
HER-2 status				
Positive	515	20 (3.9)	178	11 (6.2)
Negative	945	24 (2.5)	447	8 (1.8)
Receipt of chemotherapy				
Yes	1,076	34 (3.2)	452	14 (3.1)
No	384	10 (2.6)	173	5 (2.9)
Receipt of radiotherapy				
Yes	1,278	20 (1.6)	539	12 (2.2)
No	182	24 (13.3)	86	7 (8.1)
Receipt of endocrine therapy				
Yes	999	17 (1.7)	266	7 (2.6)
No	461	27 (5.9)	359	12 (3.3)

BCT, breast-conserving therapy; LRR, local–regional recurrence; ER, estrogen receptor; PR, progesterone receptor; TNBC, triple-negative breast cancer.

The hematological parameters included platelet count, fibrinogen, MPV, neutrophil count, monocyte count, and lymphocyte count. We used the following terms to express the correlation of hematological indices:

PF = platelet count*fibrinogen
MF = MPV*fibrinogen
PMF = platelet count*MPV*fibrinogen
FMR = fibrinogen/MPV ratio
PMR = platelet count/MPV ratio
NLR = neutrophil count/lymphocyte count ratio
MLR = monocyte count/lymphocyte count ratio
PLR = platelet count/lymphocyte count ratio

All patients signed an informed consent form upon admission. This study was approved by the Medical Ethics Committee of Shandong Cancer Hospital.

### Definition of Recurrence and Patient Follow-Up

We defined LRR as local treatment failure (including ipsilateral chest wall and skin, surgical area, and ipsilateral breast recurrence) and ipsilateral area treatment failure (ipsilateral internal mammary, supraclavicular, and axillary lymph node recurrence). There were fewer patients who had distant recurrence in our center. Distant recurrence includes nonipsilateral local recurrence and other secondary cancers (20). In our center, the definition of resection margin is to use the upper, lower, inner, and outer four margins after extended resection to represent the margin of the whole residual cavity. Re-resection is defined as secondary resection to achieve a negative margin in patients with a positive margin of first resection. A positive margin was defined as <2 mm from the surgical margin (21). The types of axillary surgery are divided into axillary lymph node dissection (ALND) and sentinel lymph node biopsy (SLNB). The subjects were followed up until February 1, 2021. The median follow-up time was 66 months (range: 6–180 months). They were followed up every 3 months in the first 2 years and every 6 months after the 3rd year.

### Treatments

For all BCT patients, we recommended radiotherapy for the whole breast at a median dose of 50 Gy, usually given in a fraction of 2 Gy/FX. Boost doses were given to the primary tumor site. The choice of chemotherapy was according to the St. Gallen consensus: patients with moderate recurrence risk received cyclophosphamide, doxorubicin (or epirubicin), and 5-Fu (CAF) regimen; patients with low risk received cyclophosphamide, methotrexate, and 5-Fu (CMF) regimen, or AC regimen; patients with high risk would receive taxane-containing regimens [AC followed by paclitaxel (P), or CAF followed by docetaxel (T), or TAC]. All the patients with positive hormone receptor status received tamoxifen (for both pre-menopausal and postmenopausal women) or aromatase inhibitors (only for postmenopausal women) for 5 years. The anti-HER2 targeted drug (Herceptin) had not officially entered the Chinese market during the study period (2006–2016) in this study group.

### Statistical Analysis

The optimal cutoff levels of PF, MF, PMF, FMR, PMR, NLR, MLR, and PLR were identified by receiver operating characteristic (ROC) curve analysis. The chi-square (*χ* ^2^) test was used to test the difference between categorical variables. The Kaplan–Meier method was used to calculate the survival curve, and the log rank test was used for univariate analysis. The Cox risk ratio model was used for multivariate analysis, and the significant risk factors in univariate analysis were used for multivariate analysis. Then, binary logistic regression was used to establish the prediction model, in which the variables came from the significant factors in multivariate analysis. A nomogram for LRR was created based on the multivariable logistic regression (*p* < 0.05). Finally, ROC curves were drawn to assess the accuracy of the prediction model, with a reasonable range of 0.5 (random) to 1.0 (perfect). The *y*-axis of the calibration curve represents the actual observed survival rate, and the *x*-axis represents the survival rate predicted by the established nomogram in the training cohort and validation cohort. All statistical data were analyzed by SPSS version 26.0 (SPSS company, Chicago, Illinois, USA) and R 4.0.3 (The R Project for Statistical Computing, www.r-project.org). *p* < 0.05 was considered as statistically significant.

## Results

### Clinicopathological Characteristics and Hematological Parameters

According to the inclusion and exclusion criteria, 1,460 patients were included in the training cohort and 625 patients were included in the validation cohort. The baseline clinicopathological characteristics in the training cohort and the validation cohort are shown in **Table 1**. In the training cohort, the median age at diagnosis was 45 years (range, 20 to 85 years), and 955 (65.4%) patients were premenopausal. A total of 427 (29.2%) patients underwent re-resection after the first positive margin, and 182 (12.4%) patients did not receive radiotherapy. There were 318 (21.8%) patients who presented with carcinoma *in situ*, including 72 with pure DCIS and 246 with DCIS and invasive ductal carcinoma. Simple carcinoma *in situ* and T1, T2, and T3 tumors were present in 76 (5.2%), 1,041 (71.3%), 333 (22.8%), and 10 (0.68%) patients respectively. A total of 1,136 (77.8%) patients were staged at N0, and the N1, N2, and N3 stages were present in 276 (18.9%), 40 (2.7%), and 8 (0.6%) patients, respectively. Among the molecular subtypes of all patients, luminal A accounted for the highest proportion (48.4%), and luminal B and triple-negative breast cancer (TNBC) accounted for relatively low proportions (7.8% and 9.2%, respectively). However, TNBC patients increased significantly in the recurrent population (31.8%). In the validation cohort, luminal A still accounted for the highest proportion (47.6%), and luminal B, HER-2 positive, and TNBC were showed in 48 (7.6%), 226 (36.1%), and 53 (8.4%) patients, respectively. A total of 492 (78.7%) patients were staged at N0, and the N1, N2, and N3 stages were present in 117 (18.7%), 11 (1.7%), and 5 (0.8%) patients, respectively.

### Recurrence Outcomes

**Figure 2:**
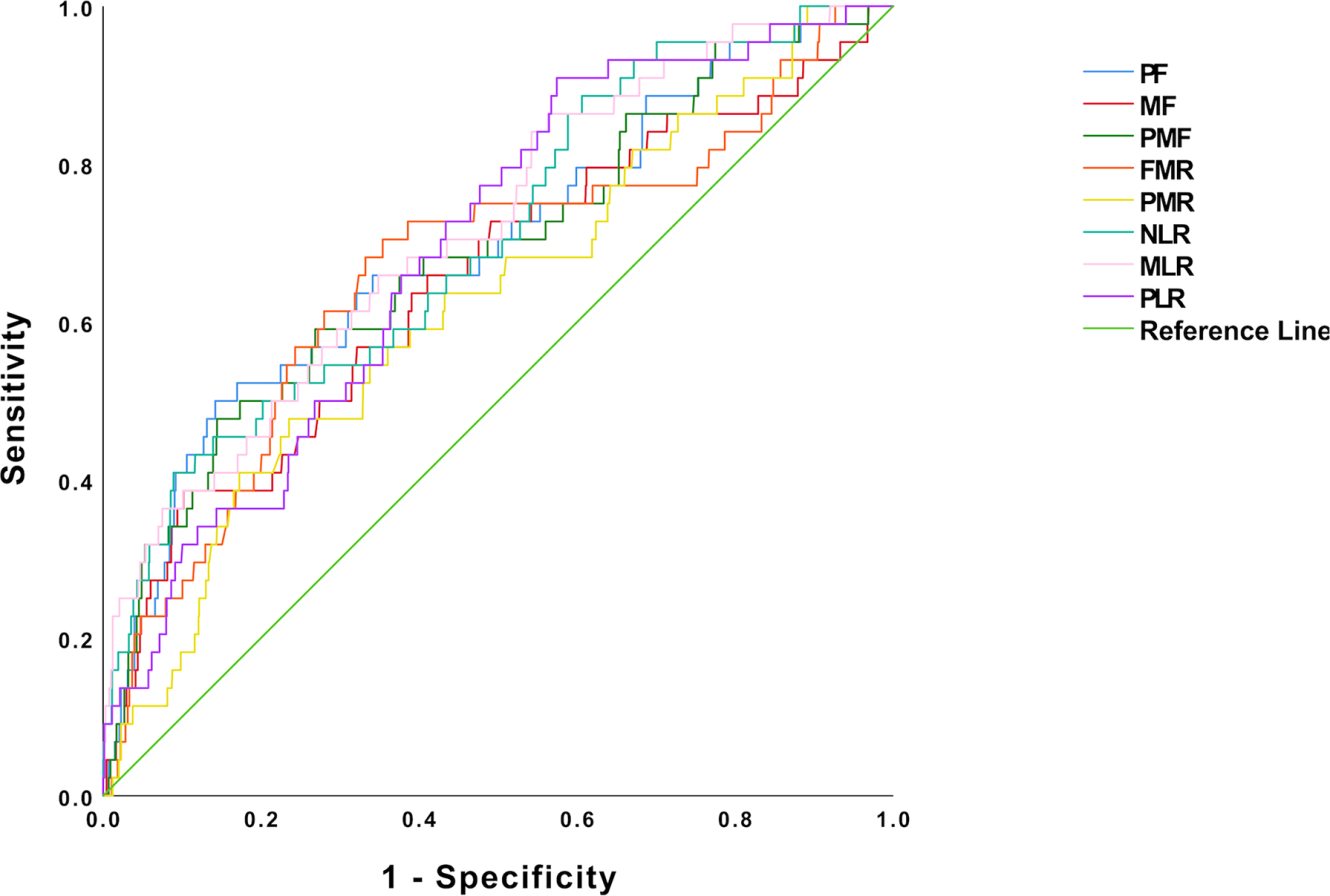
Optimal cutoff points for hematologic parameters were on with ROC curves. PF, platelet count*fibrinogen; MF, mean platelet volume*fibrinogen; PMF, platelet count*mean platelet volume*fibrinogen; FMR, fibrinogen-to-mean platelet volume ratio; PMR, platelet count-to-mean platelet volume ratio; NLR, neutrophil count-to-lymphocyte count ratio; MLR, monocyte count-to-lymphocyte count ratio; PLR, platelet count-to-lymphocyte count ratio; ROC, receiver operating characteristic.

After the median follow-up of 66 months, 44 patients (3.01%) developed LRR in the training cohort. Among the 44 patients with LRR, 23 (52.3%) patients had recurrence in the ipsilateral breast, 18 (40.9%) patients had axillary lymph node involvement, and patients rarely had chest wall and skin recurrences. **Figure 2** and **Table 2** show the ROC and cutoff values of PF, MF, PMF, FMR, PMR, NLR, MLR, and PLR of patients with breast cancer before breast-conserving surgery. The optimal cutoff point could be used for the next survival analysis.

**Table 2:** The optimal cutoff point for local–regional recurrence.

Variables	AUC	Cutoff point	*p*-value
PF	0.694	910.60	<0.001
MF	0.652	35.24	0.001
PMF	0.687	9343.58	<0.001
FMR	0.665	0.29	<0.001
PMR	0.626	27.66	0.004
NLR	0.703	4.14	<0.001
MLR	0.715	0.28	<0.001
PLR	0.691	147.67	<0.001

PF, platelet count*fibrinogen; MF, mean platelet volume*fibrinogen; PMF, platelet count*mean platelet volume*fibrinogen; FMR, fibrinogen-to-mean platelet volume ratio; PMR, platelet count-to-mean platelet volume ratio; NLR, neutrophil count-to-lymphocyte count ratio; MLR, monocyte count-to-lymphocyte count ratio; PLR, platelet count-to-lymphocyte count ratio; AUC, area under the curve.

### Univariate and Multivariate Survival Analysis

**Figure 3:**
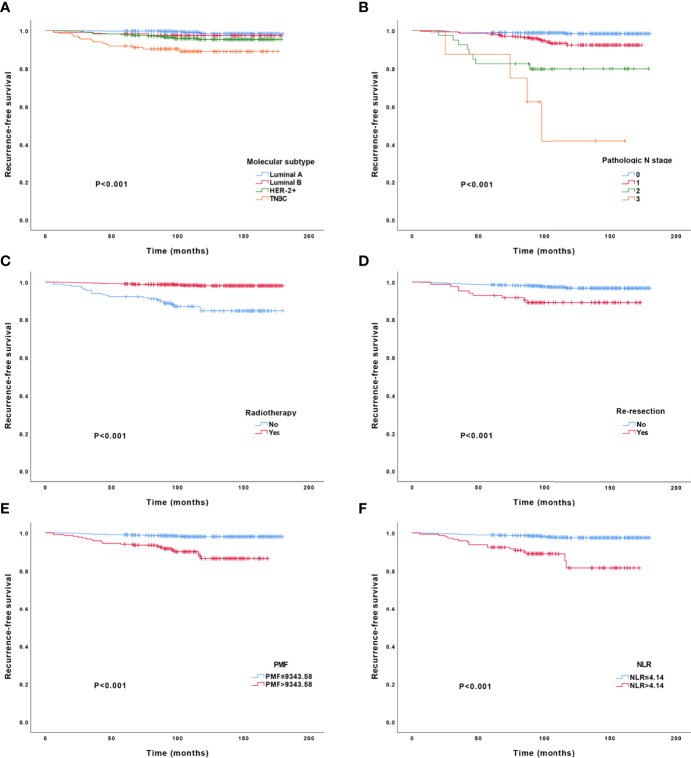
Kaplan–Meier curves for local-regional recurrence. Kaplan–Meier curves for local–regional recurrence based on molecular subtype **(A)**, pathological N stage **(B)**, radiotherapy **(C)**, re-resection **(D)**, PMF **(E)**, and NLR **(F)**. TNBC, triple-negative breast cancer; PMF, platelet count*mean platelet volume*fibrinogen; NLR, neutrophil count-to-lymphocyte count ratio.

The results of the univariate analysis of LRR in the training cohort are shown in **Table 3** and **Figure 3**, which identified the following indicators associated with LRR among patients with BCT: clinicopathological variables (age of onset, pathological stage, molecular subtype, nuclear grade, re-resection, cancer *in situ*, pathological N stage, ER, PR, radiotherapy, and endocrine therapy) and hematological variables (including PF, MF, PMF, FMR, PMR, NLR, MLR, and PLR). Further multivariate Cox regression analysis demonstrated that the independent predictive factors for LRR were molecular subtype (*p* < 0.001, HR [95% CI] = 1.904 [1.392, 2.604]), pathological N stage (*p* < 0.001, HR [95% CI] = 2.330 [1.726, 3.145]), radiotherapy (*p* < 0.001, HR [95% CI] = 0.156 [0.084, 0.292]), re-resection (*p* = 0.042, HR [95% CI] = 2.210 [1.030, 4.742]), PMF (*p* < 0.001, HR [95% CI] = 1 [1, 1]), and NLR (*p* < 0.001, HR [95% CI] = 1.316 [1.187, 1.458]).

**Table 3:** Univariate and multivariate analysis of factors for local–regional recurrence.

Factors	Univariate K-M	Multivariate Cox	
	*p*-value	HR (95% CI)	*p*-value
Age of onset	0.042	0.971 (0.939–1.004)	0.082
Pathologic stage	<0.001		0.942
Menstrual status	0.088		
Molecular subtype	<0.001	1.904 (1.392–2.604)	<0.001
Nuclear grade	0.008		0.352
Re-resection	<0.001	2.210 (1.030–4.742)	0.042
With carcinoma *in situ*	<0.001		0.390
Pathologic T stage	0.855		
DCIS only vs. pT1–3	0.896		
Pathologic N stage	<0.001	2.330 (1.726–3.145)	<0.001
Axillary surgery type	0.102		
ER	<0.001		0.161
PR	<0.001		0.663
HER-2	0.159		
Chemotherapy	0.600		
Radiotherapy	<0.001	0.156 (0.084–0.292)	<0.001
Endocrine therapy	<0.001		0.722
PF	<0.001		0.554
MF	<0.001		0.779
PMF	<0.001	1.658 (1.083–2.361)	<0.001
FMR	<0.001		0.484
PMR	0.001		0.774
NLR	<0.001	1.316 (1.187–1.458)	<0.001
MLR	<0.001		0.713
PLR	<0.001		0.762

HR, hazard ratio; CI, confidence interval; ER, estrogen receptor; PR, progesterone receptor; PF, platelet count*fibrinogen; MF, mean platelet volume*fibrinogen; PMF, platelet count*mean platelet volume*fibrinogen; FMR, fibrinogen-to-mean platelet volume ratio; PMR, platelet count-to-mean platelet volume ratio; NLR, neutrophil count-to-lymphocyte count ratio; MLR, monocyte count-to-lymphocyte count ratio; PLR, platelet count-to-lymphocyte count ratio.

### Prediction Model of the Local-regional Recurrence Nomogram

**Figure 4:**
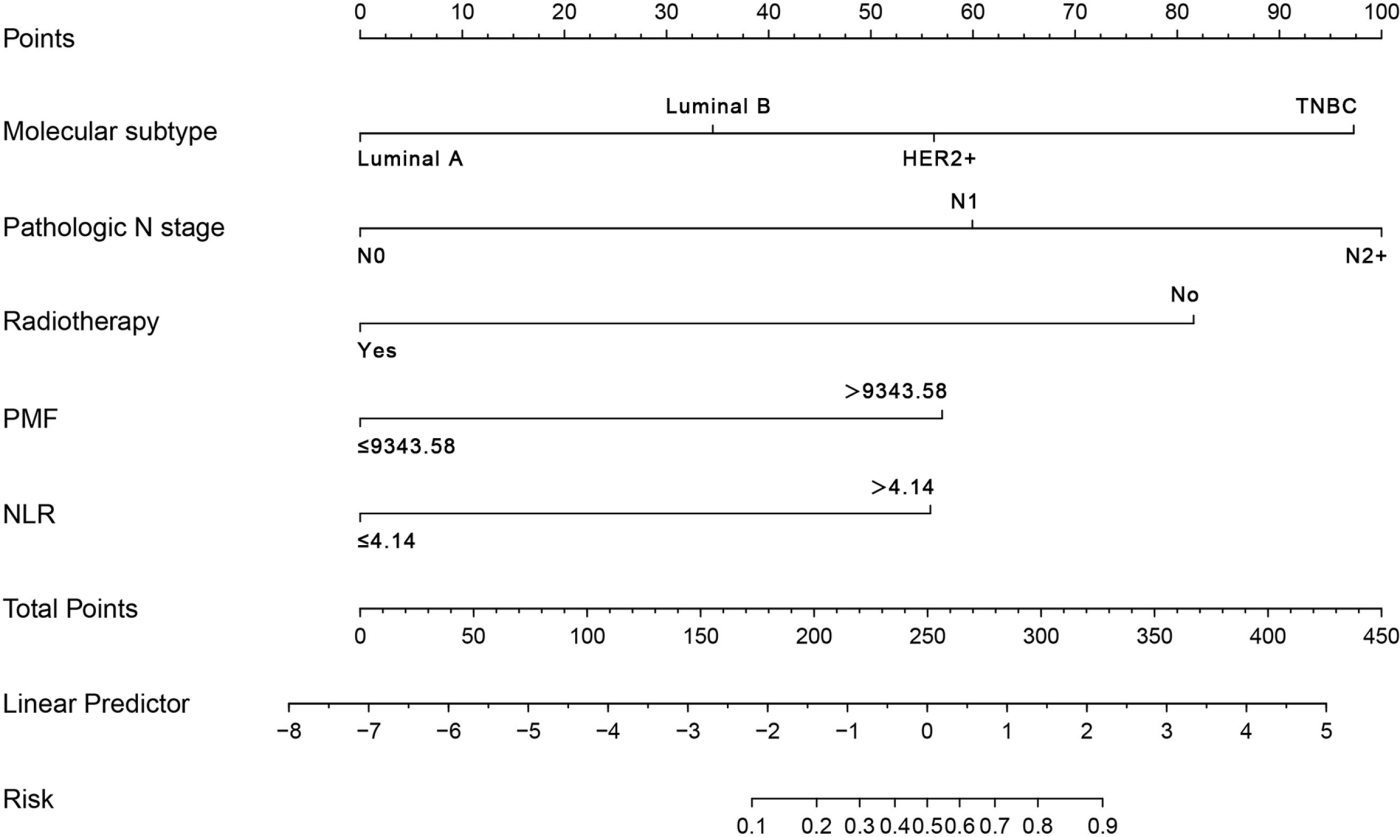
Nomogram model predicts the probability of local–regional recurrence. Points refers to point for the individual risk factor and add together to the total points. Luminal A, HER-2 (−), ER (+), PR (+) and high expression, Ki67 low expression; Luminal B, HER-2 (−), ER (+), PR (−) or low expression, Ki67 high expression; HER2+, the breast cancer of HER2 positive; TNBC, triple-negative breast cancer; N0, No positive lymph nodes; N1, the number of positive lymph nodes is 1–3; N2+, the number of positive lymph nodes is more than 3; PMF, platelet count*mean platelet volume*fibrinogen; NLR, neutrophil count-to-lymphocyte count ratio.

**Figure 5:**
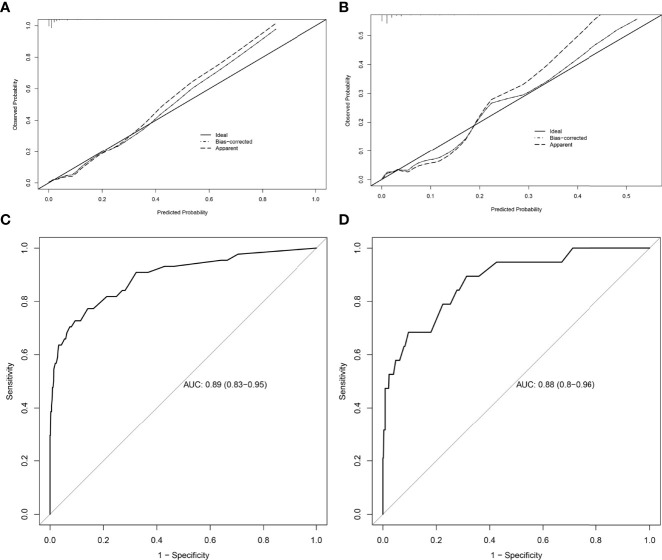
Evaluation of the LRR nomogram **(A–D)**. Calibration curves for the nomogram in the training cohort **(A)** and validation cohort **(B)**. The *x*-axis shows the predicted probability of an LRR event. The *y*-axis shows the actual LRR outcome. The discrimination assessed by ROC curves for the nomogram in the training cohort **(C)** and validation cohort **(D)**. The AUCs for LRR prediction were 0.89 (95% CI = 0.83, 0.95) in the training cohort and 0.88 (95% CI = 0.8, 0.96) in the validation cohort. LRR, local–regional recurrence; ROC, receiver operating characteristic; AUC, area under the curve.

Through univariate and multivariate analysis, a predictive model was constructed based on the independent predictors, combined with meaningful clinicopathological features and hematological parameters in multivariate analysis. The dependent variable was the incidence of LRR. After entering binary logistic regression, it was determined that pathologic N stage was the best predictor. Re-resection did not show a significant difference (*p* = 0.06, HR [95% CI] = 2.61 [0.959, 7.103]). Molecular subtype, pathologic N stage, radiotherapy, PMF, and NLR were integrated and demonstrated using a visual nomogram (**Figure 4**). The nomogram scores were given based on the weights of the independent variables in the regression model. The scale length of the nomogram variables was positively correlated with their influence on the efficacy prediction. Among all factors, pathologic N stage contributed the most to the prediction results. This was followed by molecular subtype, radiotherapy, PMF, and NLR. In pathological N stage, the high-risk segment corresponded to the high partition (scoring axis), and the low-risk segment corresponded to the low partition. The scores of all factors were added to obtain the total score perpendicular to the risk axis of LRR and the final risk of individual LRR. The nomogram of LRR showed ideal discrimination and prediction accuracy. Calibration curves for the prediction model in the training and validation cohort both demonstrated satisfactory consistency between the nomogram-predicted and actual LRR (**Figures 5A, B**). The area under the ROC curve (AUC) was 0.89 (*p* < 0.001, 95% CI = 0.83, 0.95) in the training cohort (**Figure 5C**) and 0.88 (*p* < 0.001, 95% CI = 0.8, 0.96) in the validation cohort (**Figure 5D**).

## Discussion

With the development of imaging examinations and systemic therapy, BCT has become the preferred surgical choice for patients with operable breast cancer. However, about 3% of patients still have LRR after BCT, which may be related to young age, tumor size, negative hormone receptor status, and pathologic N stage, as reported in a previous study (22–24). Moreover, the biological characteristics of breast cancer in Chinese women are different from those in Western women. The age of breast cancer patients in China is relatively young, and 50%–60% of breast cancer patients are premenopausal patients. Therefore, it is necessary to establish a practical nomogram to improve the prediction ability of LRR.

Univariate analysis showed that age of onset, pathological stage, molecular subtype, nuclear grade, re-resection, carcinoma *in situ*, pathologic N stage, ER, PR, radiotherapy, endocrine therapy, PF, MF, PMF, FMR, PMR, NLR, MLR, and PLR were related to LRR after BCT in the study. The multivariate analysis identified that independent factors for LRR included molecular subtype, pathologic N stage, re-resection, radiotherapy, PMF, and NLR. A predictive nomogram incorporating hematological parameters and clinicopathological characteristics showed ideal discrimination and consistency between the nomogram-predicted LRR and actual observation in both the training and validation cohorts.

Univariate analysis showed that TNBC had a higher recurrence rate than non-TNBC (including luminal A, luminal B, and HER-2 positivity). After multivariate adjustment, molecular subtype was still an independent factor for LRR. Our results were consistent with previous large sample studies, which proposed that IHC-based molecular subtype had significant prognostic effects. The IHC-based molecular subtype study proved that hormone receptor-negative subtypes were more likely to relapse. TNBC has strong tumor invasiveness, and hormone receptor and HER-2 receptor are negative. Due to the lack of endocrine and targeted therapeutic targets for TNBC, LRR and distant metastasis are more likely to occur in TNBC (25).

Pathologic N stage was also found to be an independent factor related to LRR. It represented axillary lymph node status. For patients with late axillary lymph node stage, on the one hand, the lymph node stage is relatively late, and there is still the risk of local residue after systematic treatment and local treatment. On the other hand, the tumors had the characteristics of near lymph node metastasis and local lymph node metastasis.

In our hospital, patients will receive re-resection due to positive surgical margins, and if the margin is positive again, mastectomy will be performed. Studies have shown that extensive intraductal carcinoma is a high-risk factor for positive margins (21). However, re-resection will destroy the integrity of the tumor. This may cause tumor cells to spread in the surgical cavity. In addition, re-resection will interfere with the definition of tumor margins, resulting in margins that are too close and even false negatives. Based on this, if only the first margin is positive, re-resection will increase the risk of local residue and recurrence. For specific types of tumors, it is more likely to need re-resection. For example, with extensive intraductal cancer, the risk of positive margins is higher, and some tumor types have the risk of false-negative margin, which is more likely to cause local recurrence. Therefore, the type of tumor requiring re-resection may also be a factor in LRR. Re-resection in multivariate analysis of Cox also showed the correlation with LRR.

Radiotherapy after BCT is the standard treatment for breast cancer in NCCN guidelines. Prospective randomized trials have shown that radiotherapy reduced the 10-year risk of any (i.e., local-regional or distant) first recurrence from 35.0% to 19.3% (absolute reduction 15.7%, 95% CI 13.7–17.7, 2*p* < 0.00001) and reduced the 15-year risk of breast cancer death from 25.2% to 21.4% (absolute reduction 3.8%, 1.6–6.0, 2*p* = 0.00005) (9, 26, 27). The overall results from these trials suggested that radiotherapy after BCT not only substantially reduced the risk of recurrence, but also reduced the risk of breast cancer death. These results suggested that the use of radiotherapy to kill tiny tumor foci in the remaining breast could reduce the risk of LRR and distant metastasis. This study also showed that postoperative radiotherapy was an independent factor for LRR. Currently, with the development of research on circulating tumor cells, studies have found that hematological parameters are important intermediaries in the occurrence and development of breast cancer. However, the detection of circulating tumor cells in clinical practice still needs more research. The literature has confirmed that common hematological parameters, such as platelets and coagulation factors, will change with the state of tumor. They are easy to obtain and can be used as an index to predict changes in tumor condition.

In the study, the PMF of the recurrence of breast cancer was obviously abnormal. Platelets have an important impact on the occurrence, development, and prognosis of tumors and can promote the direct interaction between aggrus/podoplanin and clec-2 to promote tumor growth and metastasis (13). As the main indicator of platelet activation status, MPV has also been reported to be associated with the prognosis of malignant diseases (28, 29).

In addition, some studies have found that fibrinogen levels will increase when malignant tumors or tumor-induced SIR occur, decrease after surgery, and increase again when tumor relapse occurred (30, 31). Hyperfibrinogenemia affects the prognosis of breast cancer. Tumor growth and local infiltration cause inflammation and elevate plasma fibrinogen levels, favoring stable adhesion of tumor cells and survival of metastatic embolism, which may be responsible for LRR of tumor and lymphatic metastasis (32). PMF is defined as platelet count*MPV*fibrinogen, which represents the combined effect of platelets, MPV, and fibrinogen. Some studies have shown that platelet count, MPV, and fibrinogen are changed in the recurrence and metastasis of thyroid and gallbladder cancer (33, 34). Our study also found that PMF is significantly associated with the LRR of breast cancer as an independent factor for LRR.

A prospective study conducted by the UK Biobank evaluated the correlation between prediagnostic markers of systemic inflammation and cancer risk in 440,000 participants. It proved that the ratio of inflammatory cells could be used as a biomarker of cancer risk, and it was possible to identify the disease early in the last year before clinical diagnosis (35). SIR is closely related to the prognosis of many tumors. Inflammation can promote the proliferation of cells in new plasma, stimulate angiogenesis, and reduce immunity, thereby promoting cancer recurrence and progression (19). Many studies have shown that the indicators of NLR, MLR, and PLR changed significantly in the recurrence or metastasis of breast cancer, liver cancer, and small cell lung cancer (36–38). In this study, NLR was significantly correlated with LRR as an independent factor, while MLR and PLR did not show significant correlation with LRR in multivariate analysis of LRR.

It is worth noting that age and tumor size were not independent factors for LRR in our results, which was inconsistent with previous studies. The reason may be that younger patients (less than or equal to 45 years old) are more inclined to BCT than older patients. In addition, for patients with tumors T2 or more, we can perform BCT with oncoplastic surgery, which can receive a larger margin and still keep the contour of breasts.

In this study, we established a nomogram to predict the LRR after BCT, and the AUC was 0.89, showing a satisfactory predictive effect. Additionally, despite the TNM staging system, several predictive models were explored according to inflammatory status, tumor markers, stromal tumor-infiltrating lymphocytes, gene signatures, and so on with C-indices from 0.69 to 0.77 (39, 40). Compared with these models, our predictive nomogram achieved comparative prognostic accuracy and was more economical and convenient.

However, it must be admitted that this study is a single-center retrospective study, and the number of recurrences is relatively small, so there are some uncertain biases. Therefore, the factors and prediction models of LRR need to be further verified.

In conclusion, molecular subtype, re-resection, pathological N stage, radiotherapy, PMF, and NLR are significantly related to LRR. Molecular subtype, pathological N stage, radiotherapy, PMF, and NLR can be combined to predict the LRR of patients with breast cancer after BCT. This will help clinicians to formulate individualized treatment strategies for patients after BCT according to the risk of LRR and provide patients with better treatment.

## Data Availability Statement

The original contributions presented in the study are included in the article/supplementary materials. Further inquiries can be directed to the corresponding authors.

## Ethics Statement

The studies involving human participants were reviewed and approved by Ethics Committee of the Affiliated Cancer Hospital of Shandong First Medical University. The patients/participants provided their written informed consent to participate in this study.

## Author Contributions

LS, ZY, and CL contributed to the conception and design of the study. LS organized the database. WZ performed the statistical analysis. LS wrote the first draft of the manuscript. FW, XS, and XW wrote sections of the manuscript. All authors contributed to manuscript revision, read, and approved the submitted version.

## Funding

The only funds used were those provided by the authors’ institution.

## Conflict of Interest

The authors declare that the research was conducted in the absence of any commercial or financial relationships that could be construed as a potential conflict of interest.

## Publisher’s Note

All claims expressed in this article are solely those of the authors and do not necessarily represent those of their affiliated organizations, or those of the publisher, the editors and the reviewers. Any product that may be evaluated in this article, or claim that may be made by its manufacturer, is not guaranteed or endorsed by the publisher.
